# Study on Ecosystem Service Value (ESV) Spatial Transfer in the Central Plains Urban Agglomeration in the Yellow River Basin, China

**DOI:** 10.3390/ijerph18189751

**Published:** 2021-09-16

**Authors:** Min Liu, Jianpeng Fan, Yating Wang, Chanjuan Hu

**Affiliations:** 1 College of Resource and Environment, Henan University of Economics and Law, Zhengzhou 450046, China; 20160094@huel.edu.cn; 2 Academician Laboratory for Urban and Rural Spatial Data Mining of Henan Province, Henan University of Economics and Law, Zhengzhou 450046, China; 3 Research Center for Coordinated Economic Development of the Yellow River Basin, Henan University of Economics and Law, Zhengzhou 450046, China; 4 College of Business Administration, Henan University of Animal Husbandry and Economy, Zhengzhou 450046, China; 5 Chengdu Academy of Environmental Sciences, Chengdu 610000, China; wangyt@cdaes.org.cn; 6 Institute of Geographical Sciences, Henan Academy of Sciences, Zhengzhou 450052, China; huchanjuan1981@126.com

**Keywords:** yellow river basin, urban agglomeration, ecosystem service value (ESV), ESV spatial transfer

## Abstract

Urban agglomeration is the key area to realizing regional sustainable development. Timely and accurate assessment of its ESV spatial transfer can provide a scientific basis for intercity environmental cooperation to solve transboundary environmental problems. The ESV and its spatial transfer characteristics in the Central Plains Urban Agglomeration in 2000 and 2018 were quantified by introducing the breaking point model. The findings were as follows: Firstly, taking the central city of Zhengzhou as the transferred-in area, ESV spatial transfer distributions and changes presented a trend of hinterland > metropolitan area. Secondly, the ESV spatial transfer intensity from the metropolitan area to the central city presented an increase trend, with an increase of RMB 498,400–1,053,000/km^2^, and the ESV spatial transfer intensity from the hinterland to the central city presented a decrease trend, with a decrease of RMB 15,200–814,000/km^2^ in contrast. Thirdly, a total of RMB 294.763–331.471 billion worth of ESV has been transferred, and only that worth RMB 0.534–1.716 billion reached the central city, accounting for 0.181–0.518% of the total ESV transferred and 2.760–17.482% of the central city’s ESV. Fourthly, the ESV spatial transfer radius of each city was 25.47–214.17 km, but the ESV spatial transfer range of a few cities could reach the central city. Lastly, there was inefficiency in the ESV spatial transfer only in the natural driving spatial transfer pattern due to the spatial heterogeneity of ESV distribution, and there was potential for strengthening the ecological interactions based on space guidance provided by ESV spatial transfer.

## 1. Introduction

In the new era, central cities and urban agglomerations are becoming the main spatial forms that carry development elements in China, as well as important symbols of the level of regional economic development [1,2]. In addition, urban agglomeration has become the most prominent and concentrated area where ecological protection and high-quality development interact, and its ecological support is related to the overall situation of China’s sustainable development [3,4,5,6]. The Central Plains Urban Agglomeration is one of the three major urban agglomerations in the Yellow River Basin which plays a leading role in the coordinated development of the basin, and adequate ecological service support would provide the Central Plains Urban Agglomeration with a well-developed material and environmental basis for implementing the strategy of “ecological conservation and high-quality development of the Yellow River Basin (YRB)” [7,8,9].

As an important natural resource and a socioeconomic factor of production, ecosystem services (ES) have been considered the foundation of regional development and an important indicator to measure the coordinative development between the economy and the environment since the end of the last century, and the spatial mismatch between ES supply and demand is seen as the key factor restricting and affecting sustainable regional development [10,11,12,13,14]. Unfortunately, the supply and demand of ES in different regions are always spatially mismatched due to the significant spatial heterogeneity of natural resource endowment and socioeconomic development among regions in reality [15,16,17,18].

It was found that ecosystem products could naturally move across regions in the media of water, air, soil, etc., which entailed the consequent spatial transfer of ecological services between regions [19,20,21,22,23]. Through such spatial transfer, some service functions could be transferred to areas with appropriate external conditions outside the ecosystem habitat, thus generating benefits for a larger area than the ecosystem habitat area to support socioeconomic development [4,22,24,25]. This provides a path to adjust the ES gap between supply and demand in various regions and to maintain the balance of ecosystem-derived materials and energy inside and outside each region to realize regional sustainable development goals while avoiding the degradation of ecosystems caused by an output “overload” or ecosystem service shortages in society [10,15,20,21]. In this context, valuing the ES, scientificand accurate assessment of the ESV spatial transfer, understanding the ESV spatial transfer characteristics are the basis for optimizing ecosystem service management actions, adjusting regional ecological assets, and implementing cross-regional policies for both national economic development and ecological protection [15,26,27,28]. At present, scholars at home and abroad have carried out quantitative studies on ESV and its spatial transfer in basins and cities, showing good theoretical support and important practical application value in the establishment of basin ecological compensation policy decision support [15,20,21], selection of regional sustainable development strategies [13,23,29], and planning and management of urban ES [3,30]. However, previous studies concerning both the ESV and urban agglomerations area were still concentrated in the static evaluation, and studies focusing on ESV spatial transfer of urban agglomerations are still rare [31,32,33].

With the rapid population growth and fast urbanization of the Central Plains Urban Agglomeration, the spatial imbalance of ESV has broken through the administrative boundary of a single city, undermining sustainable development of urban agglomerations. The transboundary problem can not be solved by individual cities using management style of “each fights its own battle” within the Central Plains Urban Agglomeration [34,35]. With the development of social and economic integration, how to promote the flow of ESV among regions and give full play to the overall benefits of ES has become an important issue. However, previous studies were not enough to support the Central Plains Urban Agglomeration to make ES-related intercity cooperation policies, and help the region promoting all-round cooperation in ecological co-protection, co-management and co-construction among cities.

In this paper, the ES of the Central Plains Urban Agglomeration was valued as ESV according to the land use composition, the economic value of food production services per unit of farm area and the adjusted equivalent factor table. And ESV spatial transfer characteristics in a natural state were quantitatively refined by introducing the breaking point model on the ArcGIS platform. It aims to provide a scientific reference for promoting the flow and integration of ES in the Central Plains Urban Agglomeration; to form a complementary advantage pattern of ecological sharing, ecological co-construction, and co-management; and to realize sustainable development for the Central Plains Urban Agglomeration.

## 2. Overview of Study Area

**Figure 1:**
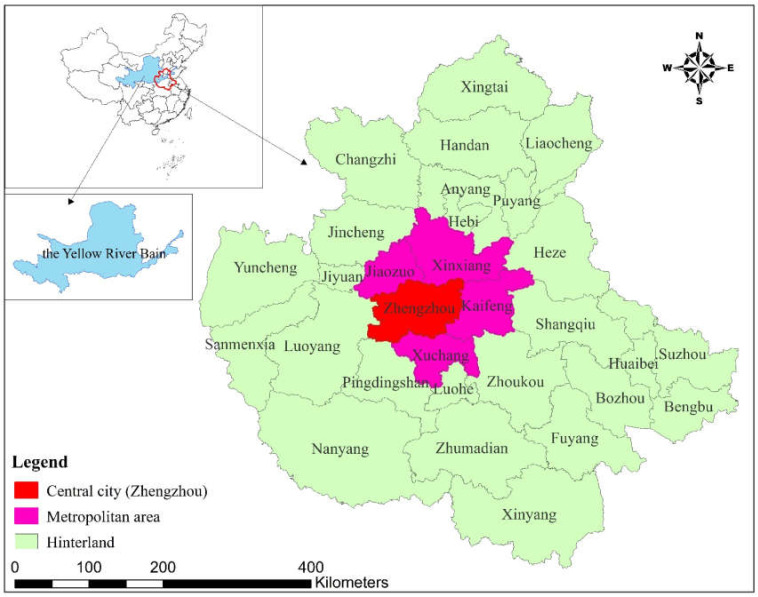
Scope and division of the Central Plains Urban Agglomeration.

With Zhengzhou as the central city; Kaifeng, Xuchang, Xinxiang, and Jiaozuo as the metropolitan area; and Xinyang, Nanyang, and 25 other cities as the hinterland (Figure 1), the Central Plains Urban Agglomeration is the largest urban group in YRB, China, with the densest population, great economic strength, rapid industrialization and urbanization, and a prominent traffic location advantage within a radius of 500 km. With a land area of 287,000 km^2^ and covering 30 prefecture-level cities in five provinces, it is an important hub “connecting the East and the West” and “connecting the North and the South” [9,35,36,37].

The Central Plains Urban Agglomeration is endowed with superior natural resources, covering three mountain ranges: the Dabie–Tongbai mountains, the Taihang Mountains, and the Funiu Mountains. It is the water source of the Middle Route Project of South-to-North Water Diversion Project and an important ecological environment protection area in China, with abundant ecosystem species. The population and economic activities are highly concentrated in cities with different scales and functions, forming the spatial structure (Figure 1) of central city–metropolitan area–hinterland [9].

At present, urban expansion and economic growth have caused transboundary environmental problems among cities in the Central Plains Urban Agglomeration, but there are no effective countermeasures or suggestions in place guiding intercity cooperation for ecological environment co-management; The Central Plains Urban Agglomeration has become one of the areas with the most prominent contradiction between humans and nature in the YRB [12,38]. Therefore, Assessing ESV spatial transfer and providing scientific basis to improve human well-beings from ecosystem-based management is of great significance to regional sustainable development [15,38,39].

## 3. Data and Methods

### 3.1. Data Sources and Processing

The spatial data selected for this study all came from the Resource and Environment Science and Data Center (https://www.resdc.cn/, accessed on 1 May 2020), including land use types, annual net primary production (NPP), and annual normalized difference vegetation index (NDVI) with a resolution of 1000 m; the data of main grain market price and grain yield per unit area are from the National Bureau of Statistics of China (http://www.stats.gov.cn/, accessed on 7 July 2020) and the National Food and Strategic Reserves Administration of China (http://www.lswz.gov.cn/html/zmhd/lysj/lsjg.shtml, accessed on 13 July 2020), respectively.

**Figure 2:**
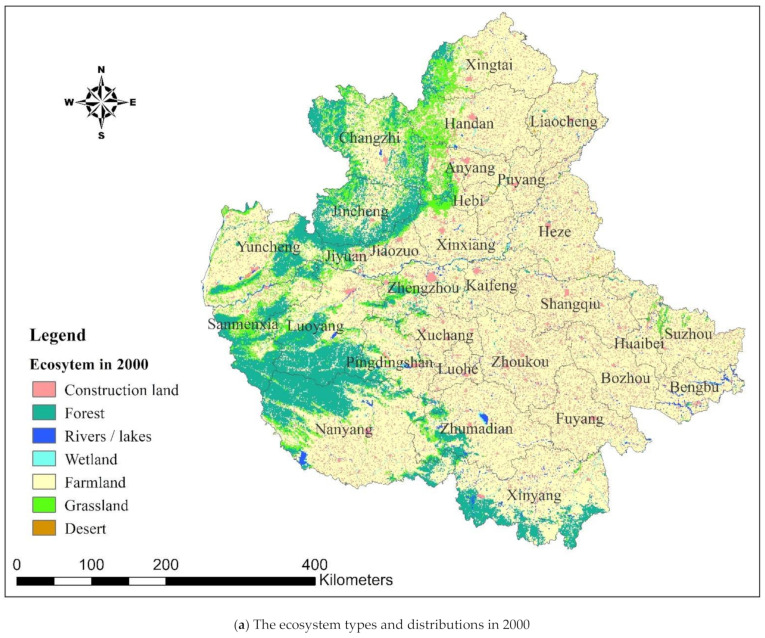

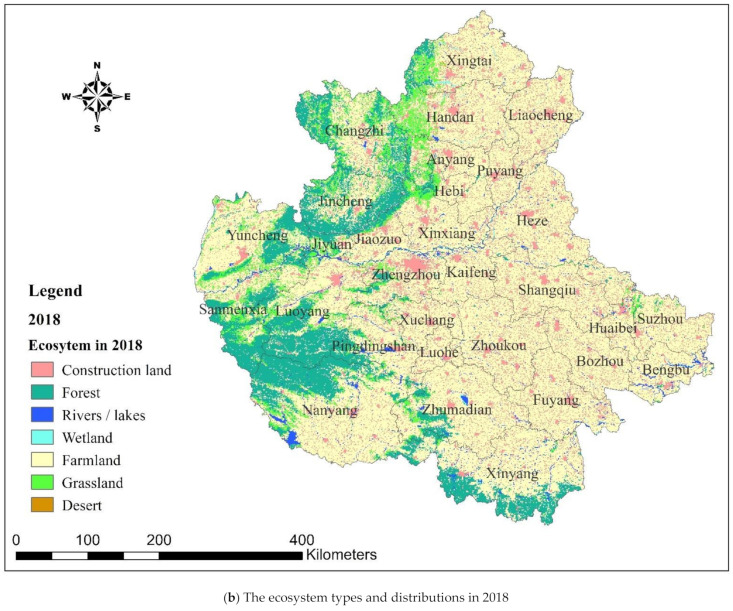
The ecosystem types and distributions obtained from the land use data of the Central Plains Urban Agglomeration in 2000 and 2018.

The ecosystem types were obtained from a reclassification of the original land use type data (Table 1), and the ecosystem distributions are demonstrated in Figure 2.

### 3.2. ESV Calculation Method

#### 3.2.1. Unit Equivalent Value

The unit equivalent value (E) refers to the “ESV Equivalent Table Per Unit Area of Terrestrial Ecosystem in China” (Table 2) [40,41]. It determined that the economic value of an ESV equivalent factor was equal to 1/7 of the national average market value of grain yield per unit area of farmland in that year. The calculation formula is as follows:

E = QF/7(1)

V_ci_ = Ea_ci_ (2)

where Q refers to the average yield per unit area of main grain in the Central Plains Urban Agglomeration from 2000 to 2018, to match the regional ecosystem characteristics of the Central Plains Urban Agglomeration and improve the accuracy of the calculation results; F refers to the average price of the main grain in China from 2000 to 2018, i.e., RMB 2497.50·t^−1^; a_ci_ refers to the ESV equivalent of different ecosystems; and V_ci_ refers to the unit area value of the type i ecosystem service of the category c ecosystem.

The ESV calculation method adopted in this paper was prompted on the base of the unit equivalent value of the national average status and was developed without considering the effect of people’s willingness to pay on setting the price of the unit equivalent value of ES. Regarding the disadvantages, it was pointed out in the original work that the ecosystem correction factor can be used to solve the price problem caused by the diversity of the ecosystems. To make the method more suitable for the study area, the following improvement was made to the method in this paper: a local correction of the average yield per unit area of main grain in the Central Plains Urban Agglomeration from 2000 to 2018 was conducted to make the method localized according to the economic value of farmland ecosystem food yield in the study area. According to the calculation method, the unit equivalent value (E) of ecological services in the Central Plains Urban Agglomeration was RMB 1894.61·hm^−2^. a^−1^, and the ESVs of ecosystems per unit area are shown in Table 3.

#### 3.2.2. Amount Calculation

After localizing the parameters, the ESV calculation adopts the quantitative remote sensing data [20,21,42]:

(3) $$\mathrm{ESV}=\overset{n}{\underset{c=1}{\sum }}V_{c}$$

where ESV refers to the total ESV; c = 1, 2, …, n refers to the type of ecosystem; and V_c_ refers to the ESV value of category c:

(4) $$V_{c}=\overset{n}{\underset{i=1}{\sum }}\overset{m}{\underset{j=1}{\sum }}R_{\mathrm{ij}}\times V_{\mathrm{ci}}\times S_{\mathrm{ij}}$$

where i = 1, 2, …, n refers to the i^th^ ecosystem service function of the category c ecosystem; V_ci_ refers to the unit area value of the i^th^ ecosystem service type of the category c ecosystem; j = 1, 2, …, m refers to the number of patches of V_ci_ in a certain area; S_ij_ refers to the area of each patch; and R_ij_ refers to the adjustment coefficient of V_ci_ in different patches, which is determined by the quality of the ecosystem. R_ij_ is the adjustment coefficient of ecosystem quality, usually characterized by the fractional vegetation cover (FVC), f, and the net primary production (NPP):

R_ij_ = (NPP_j_/NPP_mean_ + f_j_/f_mean_)/2 (5)

where NPP_mean_ and f_mean_ refer to the average values of NPP and FVC, respectively, and NPP_j_ and f_j_ are the NPP and FVC of the j^th^ patch.

f = (NDVI − NDVI_s_)/(NDVI_v_ − NDVI_s_) (6)

where f is the FVC; NDVI is the vegetation index of the plot or pixel; and NDVI_v_ and NDVI_s_ are the vegetation indexes corresponding to pure vegetation and pure soil pixels, respectively.

### 3.3. ESV Spatial Transfer Calculation Method

Based on the existing research [19,20,21], this paper introduced the breaking point formula to quantify the spatial transfer intensity and radiation radius of the ESV, and the ESV spatial transfer amount and radiation range were calculated on the ArcGIS10.1 platform.

In this paper, the ESV spatial transfer characteristics were evaluated on the basis that Zhengzhou city was considered the transfer-in area and all the cities except Zhengzhou in the Central Plains Urban Agglomeration constituted the transfer-out area.

#### 3.3.1. Spatial Transfer Radius

The ESV spatial transfer radius was calculated using the following formula:

(7) Do=Doi1+ViV0

where D_o_ refers to the radius of the ESV spatial transfer; o refers to the transfer-out area; i refers to the transfer-in area (Zhengzhou city); D_oi_ refers to the distance from the core point of the transfer-out area to the core point of the transfer-in area; and V_o_ and V_i_ refer to the value of the ES in the transfer-out area and transfer-in area, respectively.

#### 3.3.2. Spatial Transfer Intensity

The ESV spatial transfer intensity was calculated using the following formula:

(8) Ioi=VoDoi2

where I_oi_ refers to the average transfer intensity of ESV from the o region to the i region, i.e., radiation intensity.

#### 3.3.3. Spatial Transfer Amount

The ESV spatial transfer amount was calculated using the following formula:

V_oi_ = k_oi_I_oi_A (9)

where K_oi_ refers to the influencing factor of ESV in natural circulation from the transfer-out area o to the transfer-in area i, with a value between 0 and 1, and combined with the landform of the Central Plains Urban Agglomeration and the characteristics of the ecosystem, the value is 0.6 [19,20,21,32]; i refers to the type of ESV; I_oi_ refers to the radiation intensity; and A refers to the ESV spatial transfer radiation area, calculated using the buffer analysis function and overlay analysis function in the ArcGIS10.1 platform.

## 4. Results

### 4.1. ESV Amount and Distribution

#### 4.1.1. Amounts and Changes

As shown in Table 4, between 2000 and 2018, Xinyang, Luoyang, and Nanyang in the hinterland of the urban agglomeration had the largest total ESV at RMB 35.267–44.566 billion, 31.899–46.870 billion, and 52.009–64.349 billion, respectively, and also had higher ESV densities and larger scales within the urban agglomeration.

In terms of change, the cities with large decreases and the cities and counties with large increases in the total ESV were all mainly located in the hinterland, such as Luoyang, Sanmenxia, Jincheng, Xinyang, Nanyang, and Jiyuan, with changes of −31.942%, −29.328%, −23.230%, −20.866%, −19.178%, and –4.169%, respectively, while Handan, Puyang, and Liaocheng in the hinterland increased by 95.715%, 90.631%, and 89.953%, respectively.

The total ESV in the cities and counties in the metropolitan area for transfer basically remained stable, except in Zhengzhou and Xuchang. For Zhengzhou, as the central city, the total ESV in some areas increased due to the ecological protection and ecological construction along the northern Mang Mountain and the Yellow River and the construction of the Longhu water system in the Zhengdong New Area. Additionally, Xuchang’s 81.062% increase benefitted from the South-to-North Water Diversion Project.

#### 4.1.2. Density Distribution

**Figure 3:**
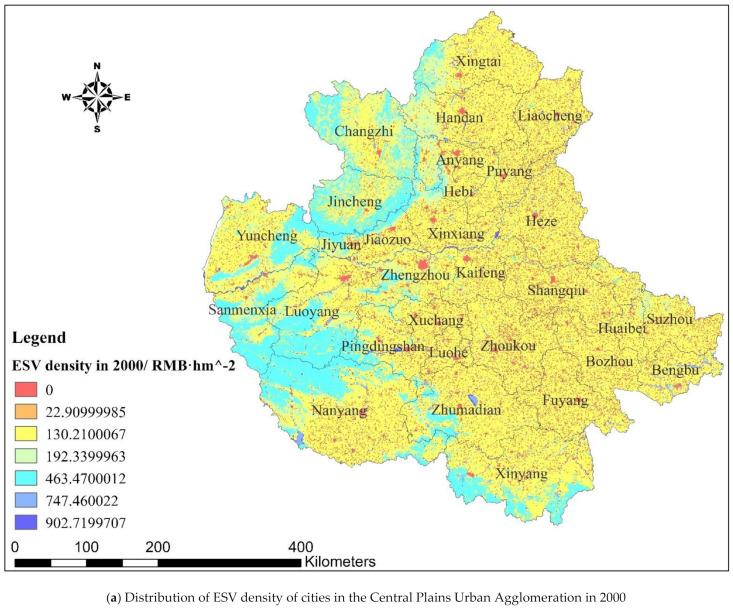

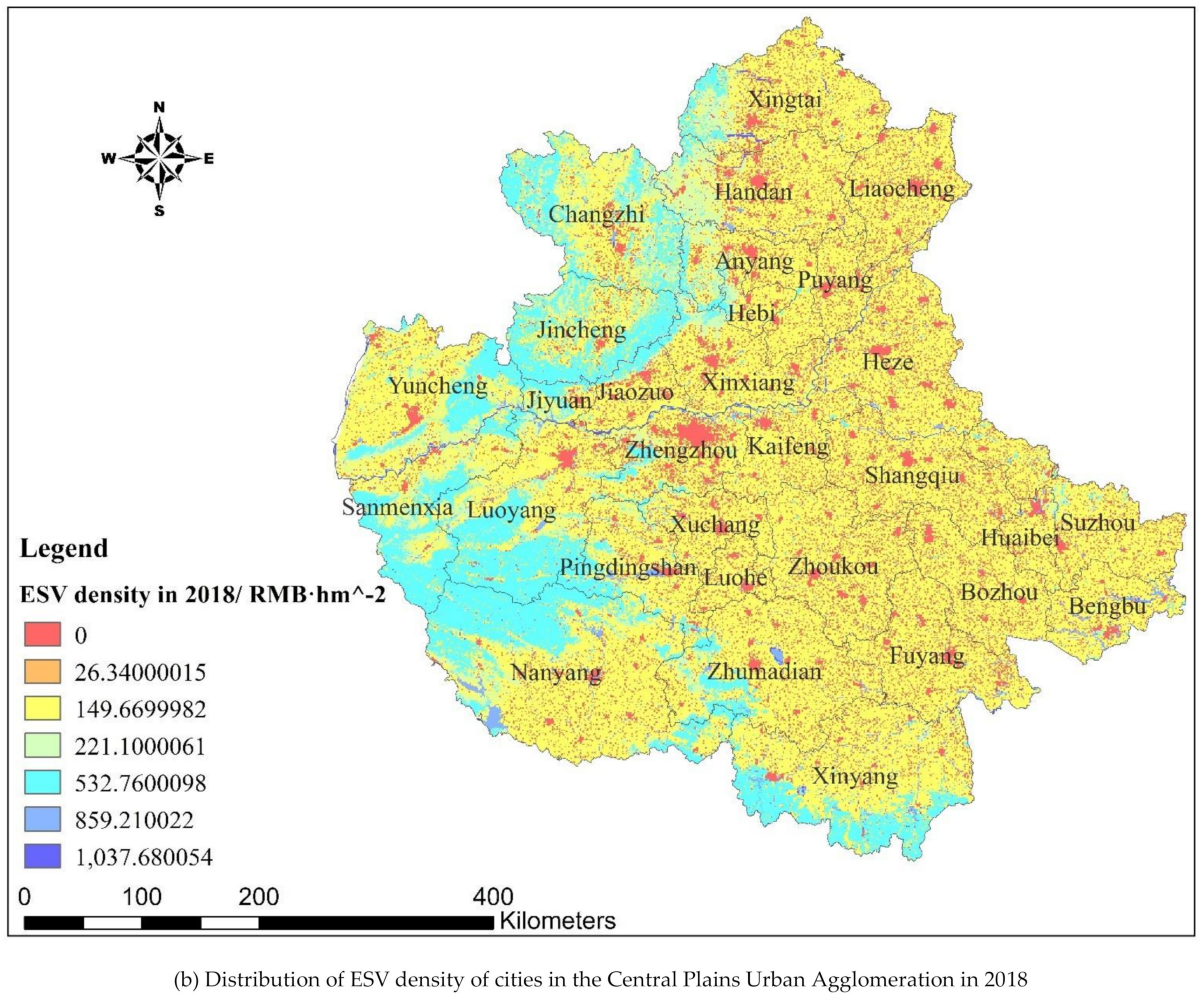

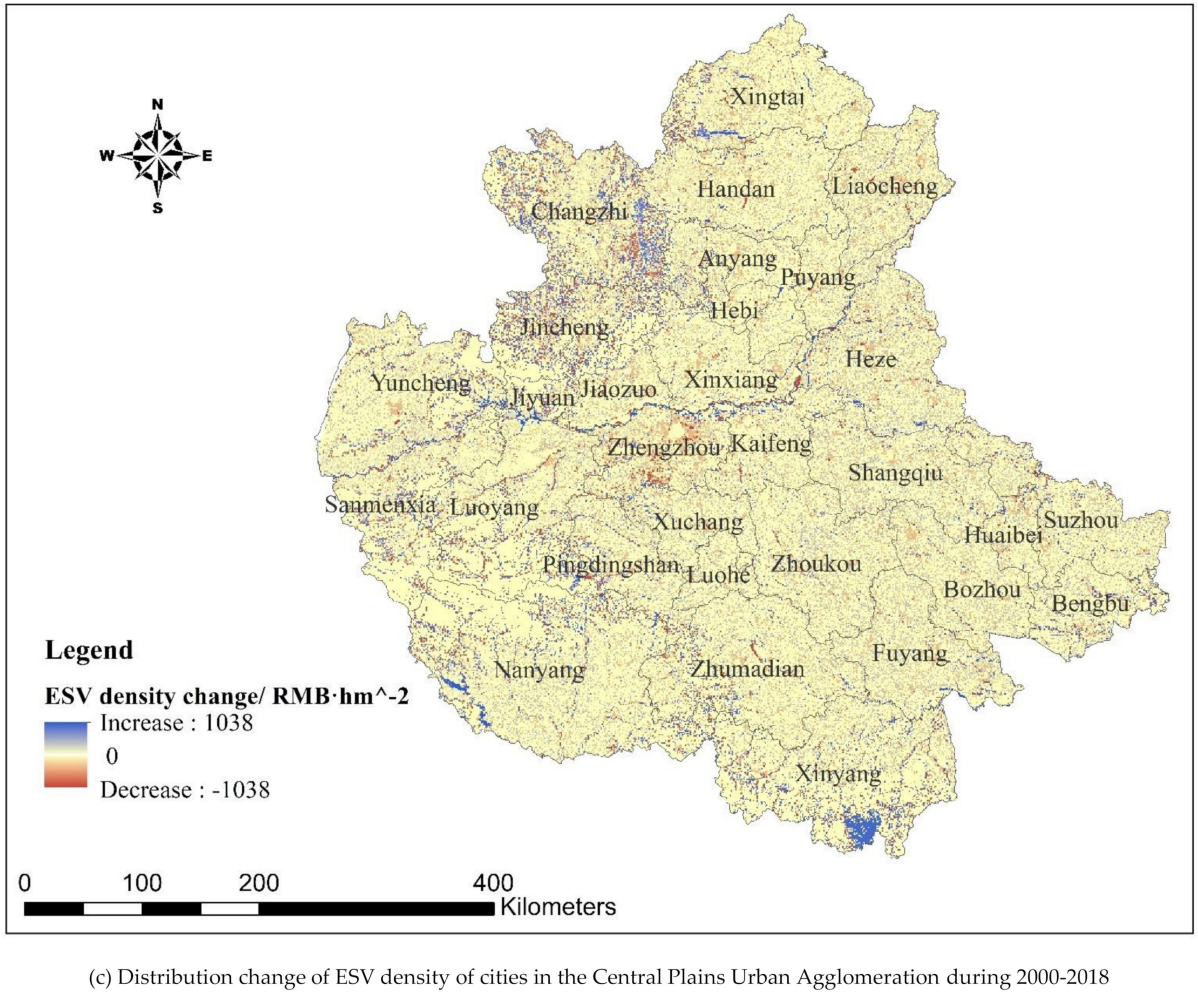
Distribution and its change of ESV density of cities in the Central Plains Urban Agglomeration in 2000 and 2018.

Using the “Natural Breaks” classification method on ArcGIS 10.1, the ESV density distribution of the Central Plains Urban Agglomeration was demonstrated. As shown in Figure 3, the ESV density distribution of the Central Plains Urban Agglomeration formed a spatial circle structure of hinterland–metropolitan area–central city from 2000 to 2018. In terms of distribution, there are obvious spatial differences in the ESV distribution in the Central Plains Urban Agglomeration, with the northwest and south of the hinterland being high ESV distribution areas, the metropolitan area being the main distribution area of medium ESV, and the central city (Zhengzhou) being the main distribution area of low ESV.

Comparing the years 2018 and 2000 in the Central Plains Urban Agglomeration, the northwest and the south of the ”hinterland” were the main areas showing an increase in ESV. The density of ESV in the “metropolitan area” remained stable, without an obvious change. The ESV of the central city (Zhengzhou) showed an overall decreasing trend.

### 4.2. ESV Spatial Transfer Intensity and Amount 

#### 4.2.1. Spatial Transfer Intensity

**Figure 4:**
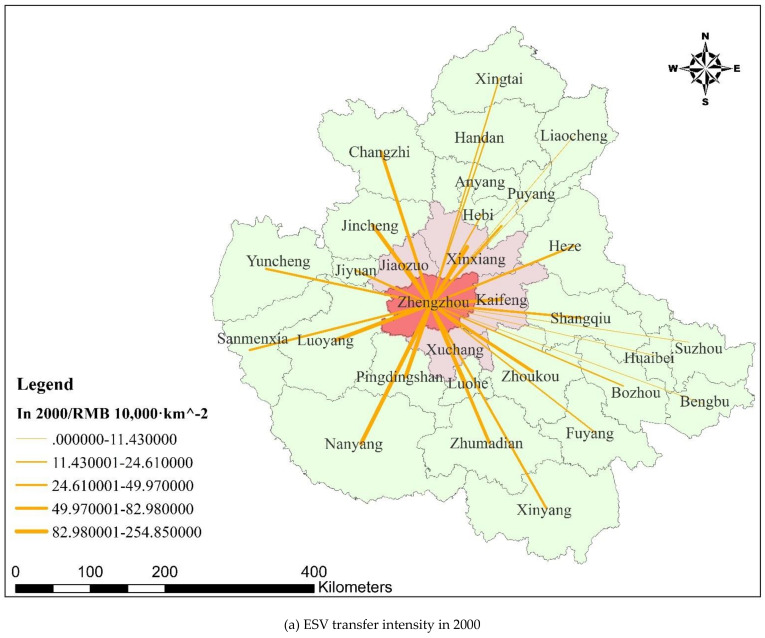

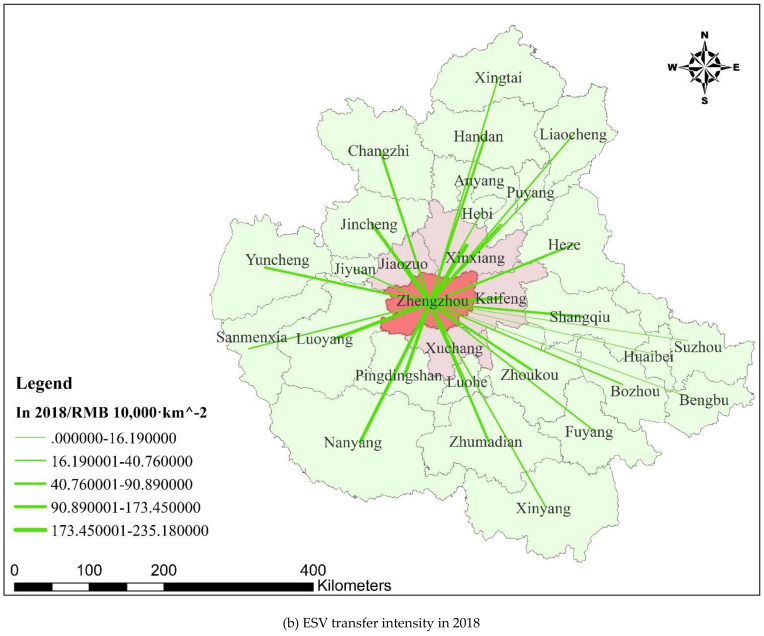

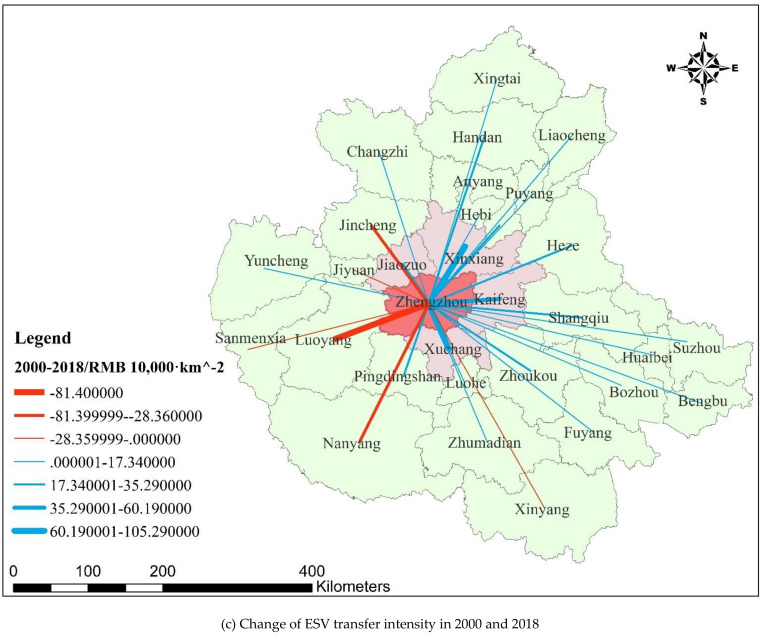
ESV transfer intensity and change of cities in the Central Plains Urban Agglomeration.

As shown in Table 5 and Figure 4, from 2000 to 2018, the spatial transfer intensity of ESV from metropolitan areas to the central city increased. The average spatial transfer intensity of ESV from Xuchang, Kaifeng, Xinxiang, and Jiaozuo to the central city has shown an overall increasing trend with an increase of RMB 498,400–1,053,000/km^2^, with Xuchang increasing the most.

The ESV spatial transfer intensity increase–decrease polarization phenomenon occurred in the cities of the hinterland. On the decrease side, from Heze, Xinyang, Sanmenxia, and Luoyang, the average spatial transfer intensity of ESV decreased by RMB 15,200–814,000/km^2^, with Luoyang decreasing the most, and the mobility of ESV to the central city became worse. On the increase side, the average spatial transfer intensity of ESV increased by RMB 23,700–352,900/km^2^ from Huaibei and Zhoukou, respectively.

The results from the ESV spatial transfer of cities in the metropolitan area suggested that ecological co-construction and cooperation in urban agglomerations is an important way to initiate the development momentum of urban agglomerations in the Central Plains Urban Agglomeration.

#### 4.2.2. Spatial Transfer Amount

From 2000 to 2018, the total amount of ESV spatial transfer in the urban agglomeration was RMB 294.763–331.471 billion, among which RMB 13.083–18.638 billion was transferred from the metropolitan area and RMB 276.123–331.471 billion was transferred from the hinterland, with Nanyang transferring the most at RMB 37.806–62.697 billion, followed by Xinyang and Luoyang (Table 6). As shown in Table 7, from 2000 to 2018, the total ESV transferred into Zhengzhou city was RMB 0.534–1.716 billion, accounting for 0.181–0.517% of the total transfer and 0.276–1.748% of the total ESV of Zhengzhou. Due to the change in the transfer radius and radiation range, the total ESV transferred into Zhengzhou decreased by RMB 1.18 billion, with a total decrease of 68.80%.

In terms of the change in transfer amount, it increased in the metropolitan area but decreased in the hinterland. However, the transfer number of the whole urban agglomeration was generally decreasing, with a total decrease of RMB 36.710 billion. The transfer amount mainly decreased in the hinterland with a total decrease of RMB 42.265 billion. Nanyang showed the largest reduction of RMB 24.891 billion, followed by Luoyang and Xinyang with decreases of RMB 22.566 billion and 16.962 billion, respectively. A total decrease of RMB 64.419 billion occurred in the three cities, accounting for 72.53% of the total decrease. The transfer amount mainly increased in metropolitan areas, with a total increase of RMB 5.555 billion, accounting for 42.46% of the total transfer amount of ESV in the metropolitan area.

Based on the results, we can draw the conclusion that natural spatial transfer may not be enough to match the ESV supply and demand, and an ecosystem conservation network composed of high-quality ES and “production base” systems in the hinterland and “ecological corridor” systems in metropolitan areas could be promoted to strengthen the ecological interaction by taking advantage of ESV spatial transfer among the hinterland, metropolitan areas, and the central city.

### 4.3. ESV Spatial Transfer Radius and Radiation Range

#### 4.3.1. Spatial Transfer Radius

The ESV spatial transfer enabled the cities and counties to transfer their ESV outside their administrative scope and increase the efficiency within the entire Central Plains Urban Agglomeration. As shown in Table 8, from 2000 to 2018, the range of the ESV spatial transfer radius of each city of the Central Plains Urban Agglomeration was 25.47–214.17 km, with Xinyang having the largest one at 180.79–214.17 km and Jiaozuo having the smallest one at 25.47–28.30 km. Compared with 2000, the ESV spatial transfer radius in 2018 showed a downward trend in all cities and counties. Among them, Xinyang decreased the most by 33.39 km, a decrease of 15.59%, followed by Sanmenxia and Nanyang, which decreased by 31.18 and 20.44 km, respectively, down by 19.61% and 13.62%, with Handan showing the smallest change with a decrease of 0.21 km, accounting for 0.16%.

#### 4.3.2. Spatial Radiation Range

**Figure 5:**
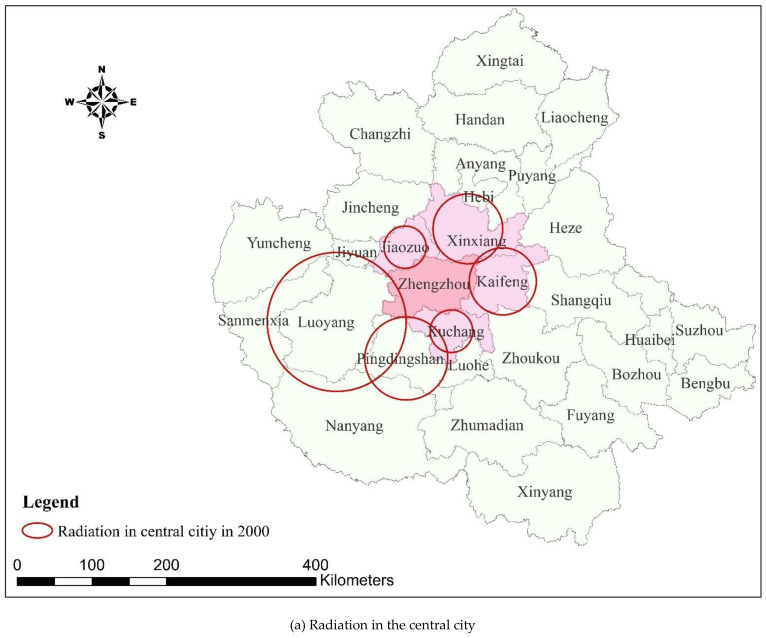

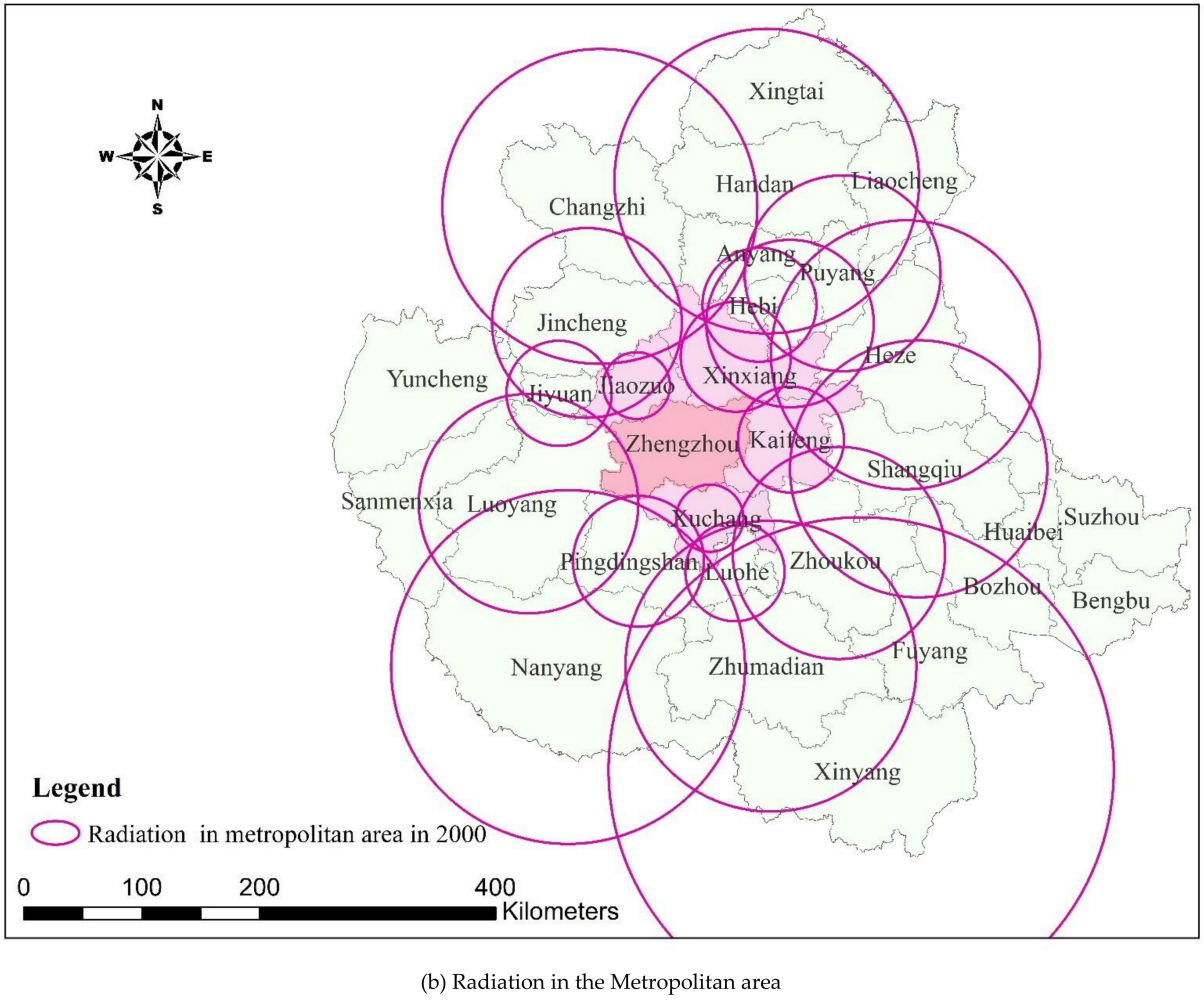

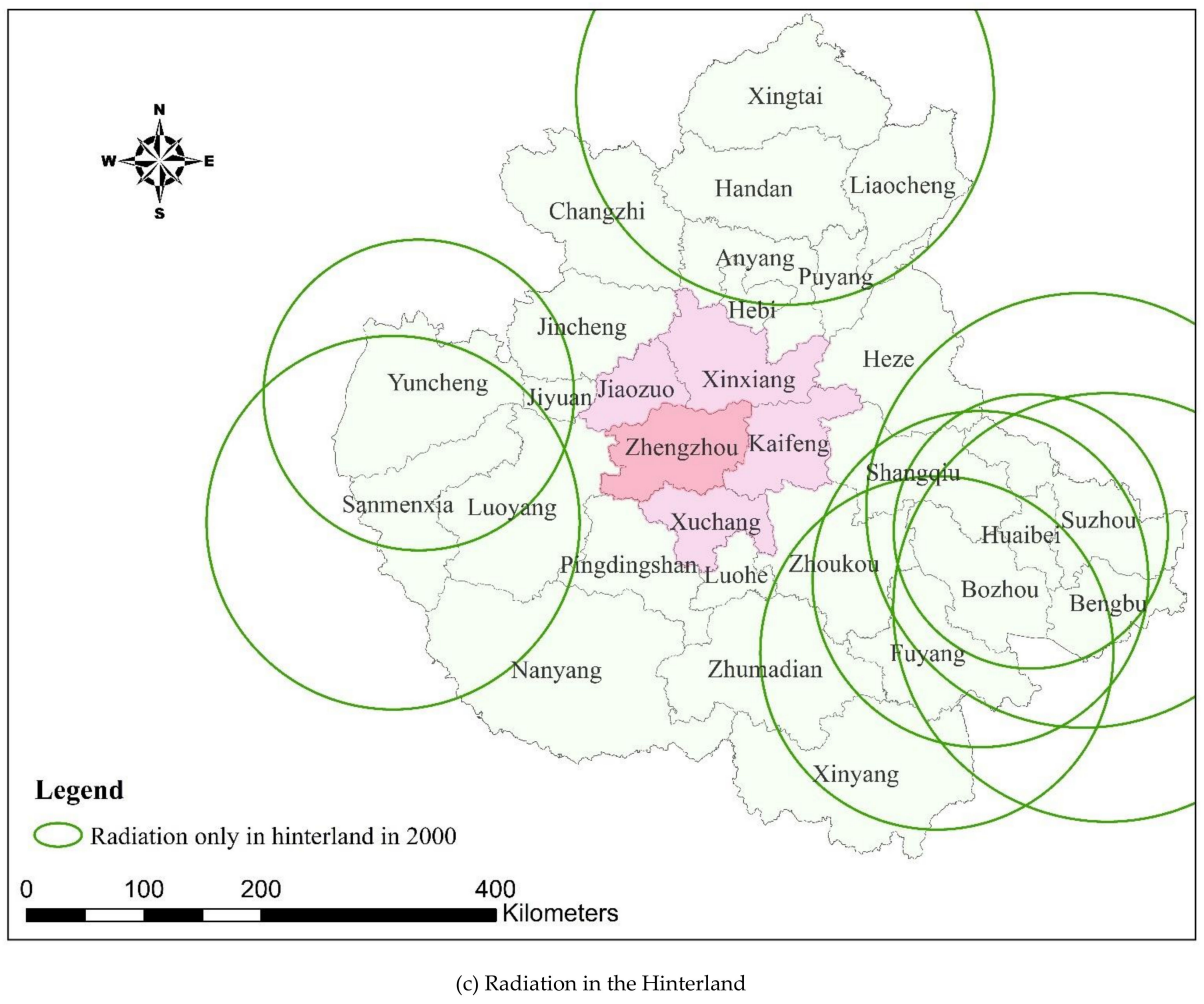

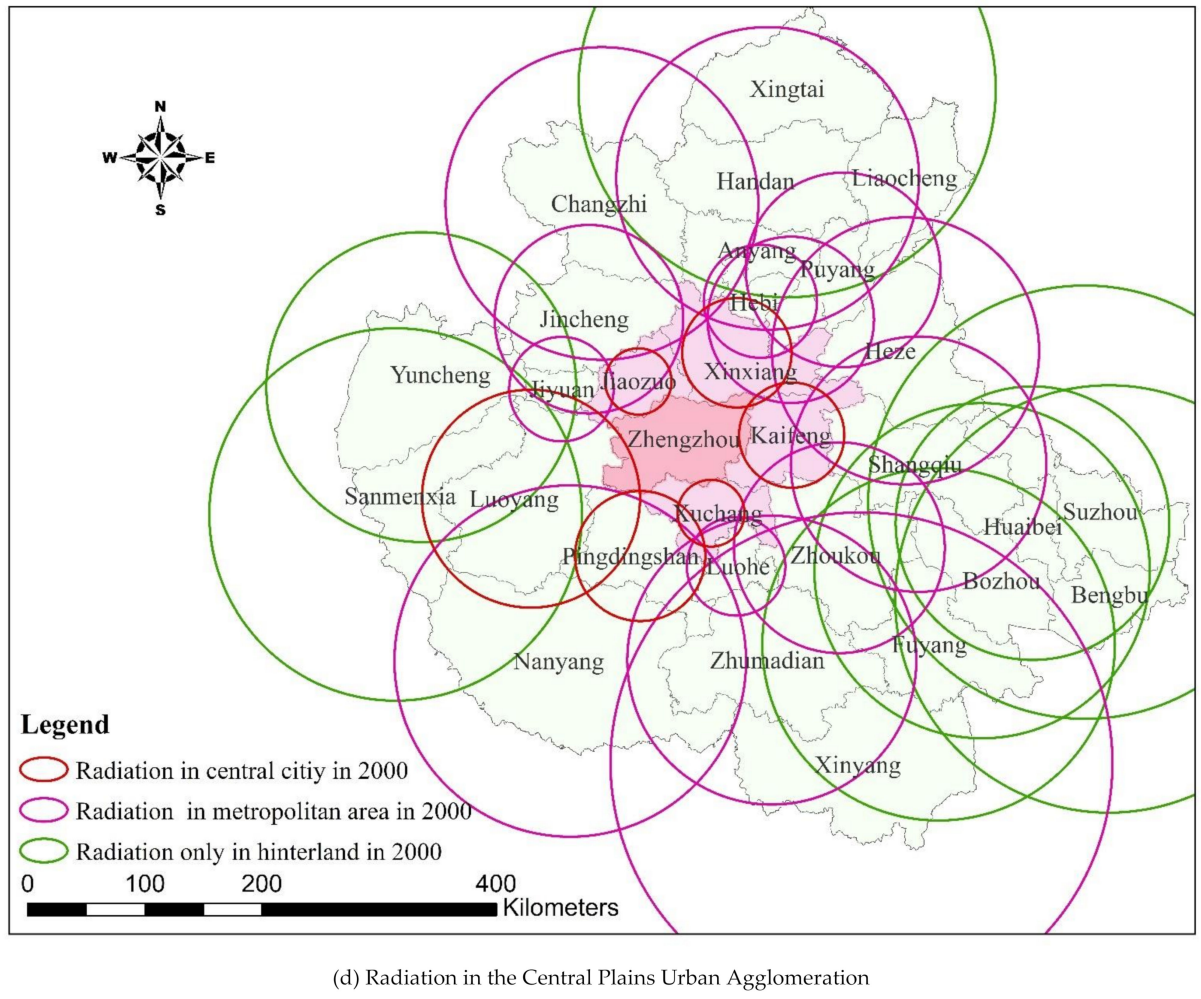
ESV spatial radiation range of cities in the Central Plains Urban Agglomeration in 2000.

**Figure 6:**
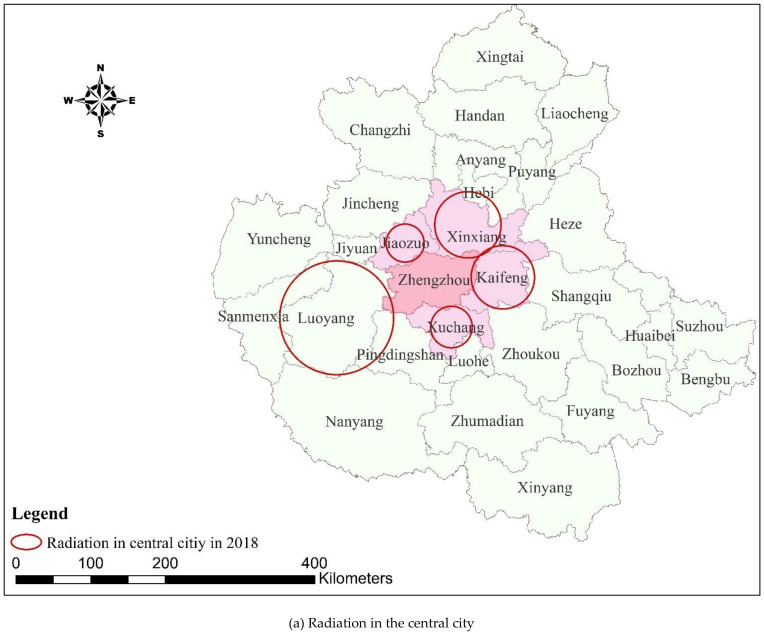

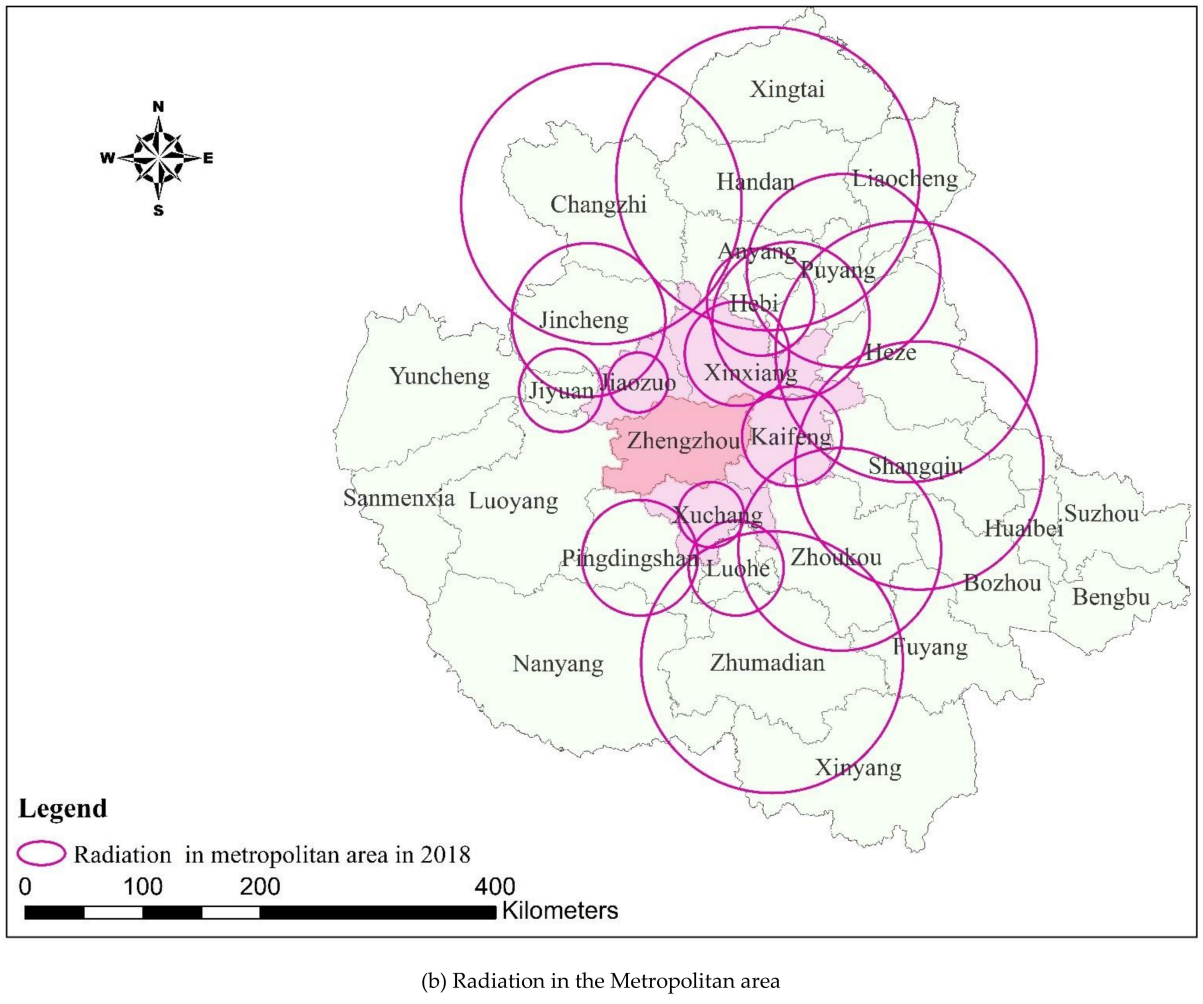

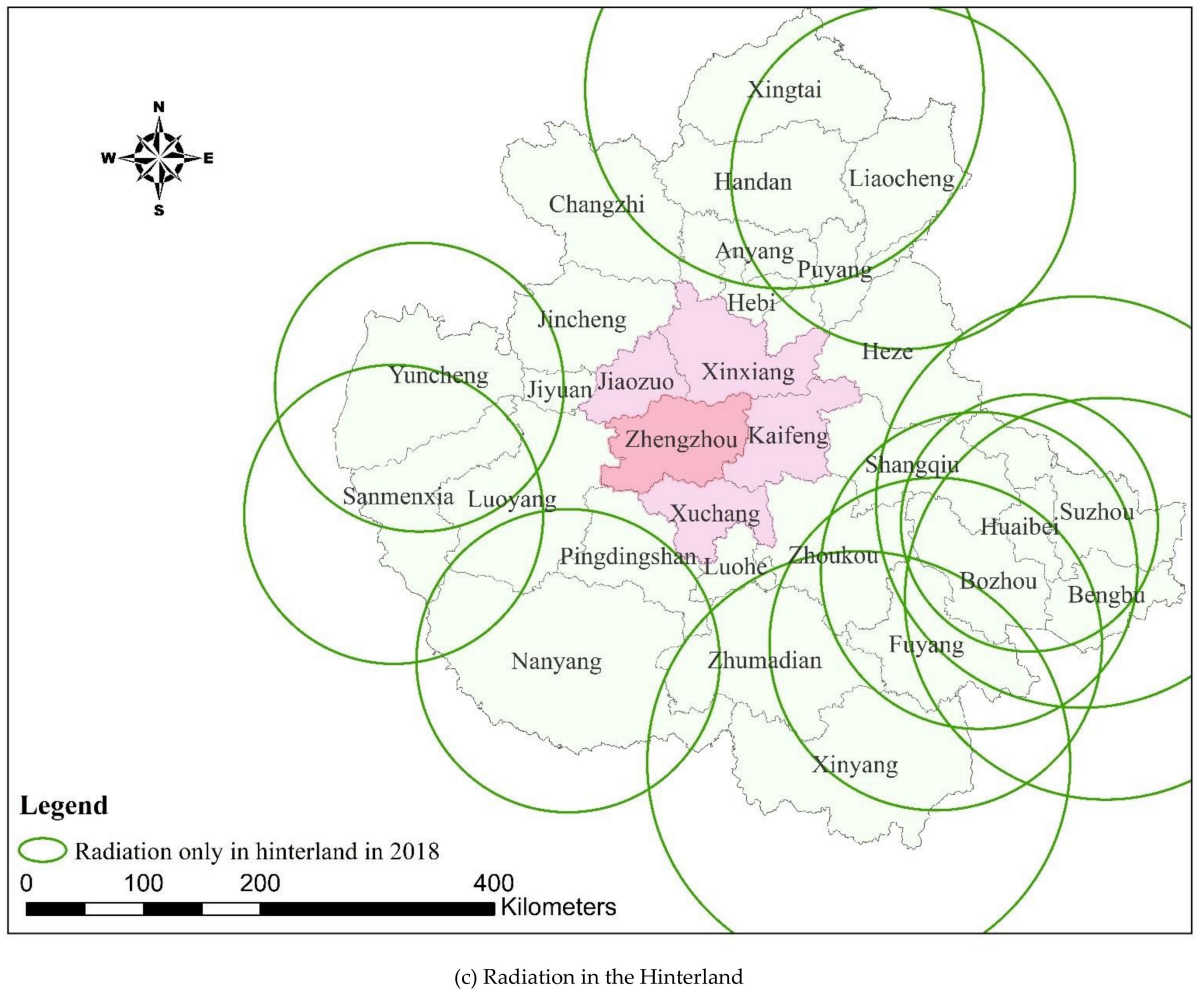

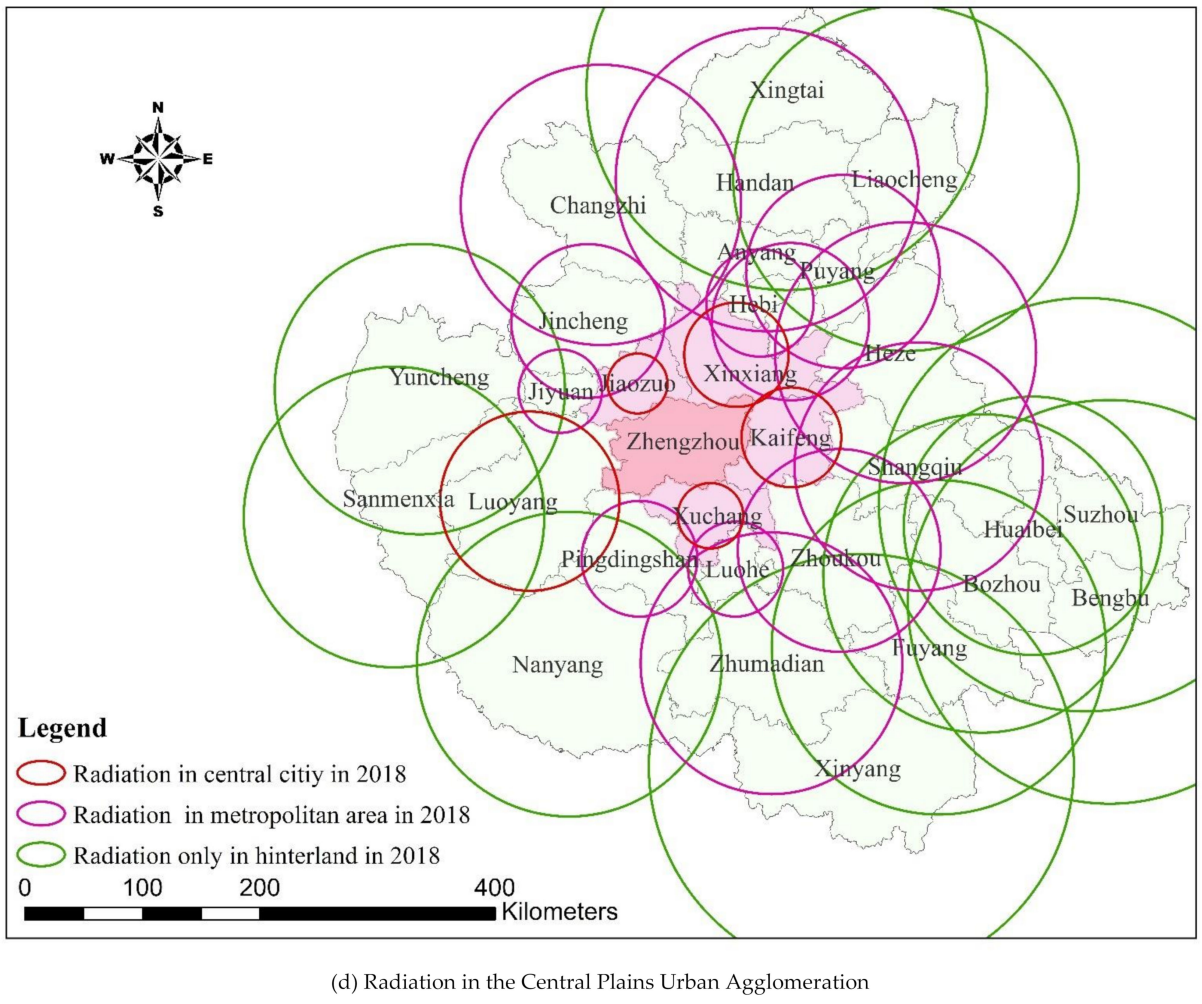
The ESV spatial radiation range of cities in the Central Plains Urban Agglomeration in 2018.

As shown in Figure 5 and Figure 6, the radiation range of ESV in the cities and counties of the urban agglomeration after spatial transfer generally reduced between 2000 and 2018. In 2000, in addition to the cities in the metropolitan area that are part of the ESV spatial transfer to the central city, there was also Pingdingshan in the hinterland. However, in 2018, only the cities and counties in the metropolitan area transferred ESV to the central city, as the radiation range of ESV spatial transfer in Pingdingshan became smaller and so could not reach the central city. In 2000, ESV was transferred to 20 cities and counties in the metropolitan area, including Handan, Liaocheng, and Changzhi in the northern hinterland; Heze, Shangqiu, and Puyang in the eastern hinterland; Xinyang, Nanyang, and Zhumadian in the southern hinterland; and Sanmenxia in the western hinterland. However, in 2018, the ESV of Sanmenxia in the west and Xinyang in the south had not radiated to the metropolitan area.

According to the GIS overlay analysis (Figure 4 and Figure 5), the ESVs of cities and counties in the urban agglomeration can exceed their administrative scope after spatial transfer and play an ecological role in urban agglomeration. Additionally, the efficiency coverage formed after the transfer of ESV has obvious spatial agglomeration characteristics and there were many intersection cities. Taking the intersection city as the core, the ESV formed an obvious spatial cluster settlement after the transfer and formed the spatial efficiency pattern of intersection city–cluster–urban agglomeration. For example, in 2000, the Anyang–Hebi–Xinxiang cluster with Hebi city as the intersection, the Jiyuan–Jiaozuo–Jincheng–Luoyang cluster with Jiyuan as the intersection, the Xuchang–Luohe–Pingdingshan cluster with Luohe as the intersection, and the Kaifeng–Zhoukou–Heze–Shangqiu cluster with Shangqiu as the intersection. This efficiency pattern can provide clear spatial guidance for the construction of the ecological network of the Central Plains Urban Agglomeration and for coordinated ecological governance/management.

Based on the radiation range of ESV spatial transfer, it indicated that the concepts of a “big region” and a “big environment” view are needed to help people to establish regional collaborative governance mechanisms to integrate ecosystem-based management among cities for solving transboundary environmental problems caused by urban expansion and the spatial heterogeneity of natural endowments.

## 5. Discussion

### 5.1. Implications

Transboundary environmental problems are common problems of urban agglomerations, and they need all-round cooperation among cities in the region to solve these problems [15]. At present, transboundary environmental problems of the Central Plains Urban Agglomeration have become an obstacle to further development, and the existing eco-management pattern conducted by individual cities is insufficient. It is urgent to promote the integration of environmental protection and governance among cities in the region. In this context, ES spatial transfer provides a path to strengthen the co-construction and sharing among cities in the region to release the development momentum of urban agglomerations. However, based on the results, the ESV amount and density of a given city is limited by the ecosystem type and its habitat distribution, which directly influences the ESV spatial transfer amount, intensity and radius among cities, affecting intercity ecological cooperation in the region, causing low efficiency of ES spatial transfer between cities under natural conditions. Diversified cooperation mechanisms should be established to promote the ES flow and integration to strengthen intercity cooperation.

For the work mechanism at intercity ecological construction and protection, we pro-pose to establish a four-level Joint Meeting mechanism among central city, metropolitan area, hinterland, and urban agglomeration to form complementary work programs, to fully implement ecological environmental protection plans and policies conducted by the nation, the basin, and individual cities, and to solve transboundary environmental problems.

In terms of ecological construction, based on the intercity ecosystem status and its ES spatial transfer characteristics, we propose to build a sustainable network of ES “production”-”flow”-”consumption”, which composites of high-quality ES “pro-duction base” systems for ES “supply” in the hinterland, ecological corridor systems for ES “flow” in metropolitan areas, and green infrastructure systems for ES “consumption” in built area inside the city. The system could be considered as nature’s porters to help ES spatial transfer, guiding the cities to perform regional and comparative advantages in ES management and integrate regional ecological resources to achieve intercity cooperation.

For institutional improvement, we propose to establish intercity ecological compensation policies among cities based on the ESV spatial transfer to ensure sustainable ES man-agement through sustainable land use management in ES surplus cities, enabling the ESV transfer sustainably between ES “supply” city and ES “consumption” city to achieve regional sustainable development.

### 5.2. Contributions and Limitations

At present, studies have connected ESV spatial transfer with interregional ecological linkage analysis and policy making at ecological cooperation in regions [15]. Some of these studies have used ESV spatial transfer analysis as a scientific basis for ecological compensation policy decision support to establish a cooperation pattern between the upstream cities and downstream cities in watershed regions [20,21]. Some studies applied ESV spatial transfer to reveal the emergence of transboundary ecological and environmental problems and seek solutions [15]. In addition, some urban ecological planning and managements were made according to the ESV intercity spatial transfer for human well-being from ecosystem-based management [3,28,43,44]. Urban agglomeration area usually facing transboundary environmental problems because of its remarkable population growth and urbanization. There are urgent demands for ecological integration and cooperation based on ESV spatial movements. However, studies concerning ESV spatial transfer and urban agglomeration area were still rare. This study quantitively evaluated the ESV spatial transfer characteristics of the urban agglomeration area and identified the ESV spatial transfer structure inside Central Plains Urban Agglomeration, and that can provide a scientific basis for intercity cooperation to support all-round environmental policy decision making and solve the transboundary environmental problems in urban agglomeration regions.

In this study, to value the ES is the premise of the ESV spatial transfer calculation. But the value of ES is difficult to be measured accurately, and the uniform criterion, principle, and methods of ES evaluation is lacking until now. In this study, an expert knowledge-based “equivalent value” method was used to convert different types of ESs into monetary values [25,30]. Although it still needs further refinement in this value conversion method [41,45,46], the monetized ES have been improved to be both convenient and analytically effective through validation by interviews of local experts across China [20,21,40,47]. In this study, we evaluated the monetary value of ESs based on the land use /cover, major grain-producing areas in the Central Plains Urban Agglomeration, and China’s main grain prices from 2000 to 2018, and the results were consistent with that of Chen [47], Yang [31], and Wang [32].

In addition, subject to the implementation of the minimum purchase price policy in major grain-producing areas, China’s main grain prices have not changed much since 2004, and this led to the ESV during the research period being relatively stable, resulting in the ESV in the study area mainly changing with the change in urban land use type. Therefore, in the results, it was found that the distribution and change of the ESV were mainly driven by natural resource endowment, social and economic development, and management policy differences. Firstly, there were differences in the types of natural ecosystems and their distribution. The northwest and the southern parts of the “hinterland” mainly contained forest, wetland, water systems, and other ecosystems, with a high ESV per unit area, making the region the main distribution area of high ESV. There were more agricultural lands in the metropolitan area, with a medium ESV per unit area and a basically stable scale of agricultural land, which was also the basis for the stability of the ESV in the metropolitan area. The central city (Zhengzhou) consisted of highly concentrated “construction land area”, with a low ESV per unit area. Secondly, there were differences in the urbanization levels of cities in the Central Plains Urban Agglomeration. With the evolution of the spatial circle structure of the Central Plains Urban Agglomeration, the central city (Zhengzhou), the regional central city, and the surrounding towns in its hinterland have gradually entered the stage of polarization development [34,35], and the social and economic development level, urban function, and intensity of urban development and construction of each city have initially formed a spatial circle structure of central city > metropolitan area > hinterland. Moreover, urban expansion and economic growth turns a lot of land types into construction land, especially farmland, forest, river/lakes, wetland…, these land types are high-ESV land, the increase in urban construction land has led to resulting in the decrease in high-ESV land, bringing about the spatial differences in ESV change and making the central city the main ESV reduction area and one with a lower ESV density. Finally, there were differences in the spatial protection policies within urban agglomerations. In recent years, China has made unprecedented efforts to protect the ecological environment of ecological spaces. In Central Plains Urban Agglomeration, the government has made a series of eco-plans and a series of ecological restoration and construction projects to protect the mountains, forests, farmland, river/lakes, grassland. With the implementation of these plans and projects, the ecosystem quality has been improved as well as the ESV density. Such initiatives include the Tongbai–Dabie Mountains Ecological Barrier Area, the Funiu Mountains Ecological Barrier Area, the Taihang Mountains Ecological Barrier Area, the Ecological Corridor in the Middle Route of the South-to-North Water Diversion Project, the Ecological Corridor Along the Middle and Lower Reaches of the Yellow River, the Ecological Corridor of the Old Course of the Yellow River in the Ming–Qing Dynasties, and the “Three Barriers and Four Corridors” Ecological Space of Ecological Economic Corridor Along the Huaihe River. The “Three Barriers and Four Corridors” Ecological Space has become the main distribution area of high ESV. As the main distribution areas of ecological space, the northwest, south, and central parts of the hinterland have also become the main growth areas of ESV. In brief, the natural endowments, urbanization state, and management policies varied in the different areas, which resulted in the spatial differences in the ESV amount, distribution, and demands in the Central Plains Urban Agglomeration. The spatial mismatch caused by these factors between the high ESV density area, also known as the high ESV “supply” area, and the low ESV density area Zhengzhou, also known as the high ESV “demand” area, created a realistic need for an integrated management of ES in the urban agglomeration based on ESV spatial transfer.

## 6. Conclusions

This paper draws the following conclusions:

(1) The ESV distributions presented a trend of hinterland > metropolitan area > central city due to the spatial heterogeneity of natural resource endowment and socioeconomic development level in the Central Plains Urban Agglomeration. Additionally, the ESV could naturally be transferred from the hinterland, the main ESV transferred-out area showing increases, and the metropolitan area to the central city. The distributions of transferred ESV presented a trend of hinterland > metropolitan area.
(2) The spatial transfer intensity of ESV from the hinterland to the central city was reduced, indicating a “weakening” ecological correlation between the hinterland and the central city. The spatial transfer intensity of ESV from the metropolitan area to the central city was increased due to the preliminary integration of ecological protection and governance among cities in the metropolitan area, which could ensure the central city benefit from cities in this region in terms of ES.
(3) Spatial transfer was a pathway of ES delivery from the hinterland and the metropolitan area to the central city. But only very small part of ESV was delivered under natural conditions in this paper. There is still great potential for strengthening all-round intercity cooperation at the ecological protection and governance among the hinterland, the metropolitan area, and the central city, to achieve sustainable development of the urban agglomeration area.
(4) The ESV spatial transfer radius and the radiation range of each city was tended to shrink. The ESV spatial transfer radius of most cities in the hinterland and the metropolitan area could not reach the central city, resulting in the inefficiency of the ESV spatial integration within the Central Plains Urban Agglomeration.
(5) According to the characteristics of ESV spatial transfer, some works could be suggested to accelerate the spatial movement of ESV as well as the ecosystem-derived material and energy to provide an ecological path, solving the transboundary problems and increasing the development momentum of the Central Plains Urban Agglomeration: Firstly, the concepts of a “big region” and a “big environment” view should be established. Secondly, the intercity integration of ecological protection and governance should be promoted, especially a long-run administrative mechanism should be promoted to strengthen all-round cooperation among cities. Thirdly, an ecosystem network consisting of high-quality ES “production base” system, well connected “ecological corridor” system and feasible ES “consumption” infrastructures should be built based on current “conservation land” system and ecological infrastructures in prospective to provide carrier for ESV transfer.

## Figures and Tables

**Table 1:** Ecosystem types and reclassification of original land use data.

Ecosystems	Land Use Types of the Original Land Use Data
Forest	Broad-leaved evergreen forests, deciduous broad-leaved forests, evergreen coniferous forests, deciduous coniferous forests, mixed coniferous and broad-leaved forests, evergreen broad-leaved shrub forests, deciduous broad-leaved shrub forests, evergreen coniferous shrub forests, arbor garden, shrubby garden, arbor green space, shrub green space, sparse forests, sparse shrubbery
Grassland	Water meadow, grassland, thick growth of grass, herbaceous green space, sparse grassland
Farmland	Paddy field, dry land
Wetland	Wetland, forest swamp, shrub swamp, herbaceous swamp
Rivers/lakes	Lake, reservoir/pond, rivers, canal/ditch
Desert	Moss/lichen, bare rock, bare soil, desert/sand, saline alkali land
Construction land	Residential land, industrial land, traffic land, mining area

**Table 2:** ESV equivalent table per unit area of terrestrial ecosystem in China [40,41].

ES		Forest	Grassland	Farmland	Wetland	Rivers/Lakes	Desert	Construction Land
Supply services	Food production	0.33	0.43	1.00	0.36	0.53	0.02	0.00
	Raw material production	2.98	0.36	0.39	0.24	0.35	0.04	0.00
Regulatory services	Gas regulation	4.32	1.50	0.72	2.41	0.51	0.06	0.00
	Climate regulation	4.07	1.56	0.97	13.55	2.06	0.13	0.00
	Hydrological regulation	4.09	1.52	0.77	13.44	18.77	0.07	0.00
	Waste disposal	1.72	1.32	1.39	14.4	14.85	0.26	0.00
Support services	Soil conservation	4.02	2.24	1.47	1.99	0.41	0.17	0.00
	Biodiversity	4.51	1.87	1.02	3.69	3.43	0.40	0.00
Culture services	Aesthetic landscape	2.08	0.87	0.17	4.69	4.44	0.24	0.00

**Table 3:** ESVs of different ecosystems per unit area in the Central Plains Urban Agglomeration (RMB ·hm^−2^.a^−1^).

Ecosystems	Food Production	Raw Material Production	Gas Regulation	Climate Regulation	Hydrological Regulation	Waste Disposal	Soil Conservation	Biodiversity	Aesthetic Landscape
Forest	625.22	5645.94	8184.72	7711.06	7748.95	3258.73	7616.33	8544.69	3940.79
Grassland	814.68	682.06	2841.92	2955.59	2879.81	2500.89	4243.93	3542.92	1648.31
Farmland	1894.61	738.90	1364.12	1837.77	1458.85	2633.51	2785.08	1932.50	322.08
Wetland	682.06	454.71	4566.01	25,671.97	25,463.56	27,282.38	3770.27	6991.11	8885.72
Rivers/lakes	1004.14	663.11	966.25	3902.90	35,561.83	28,134.96	776.79	6498.51	8412.07
Desert	37.89	75.78	113.68	246.30	132.62	492.60	322.08	757.84	454.71
Construction land	0.00	0.00	0.00	0.00	0.00	0.00	0.00	0.00	0.00

**Table 4:** The ESV amount and change rate of cities in the Central Plains Urban Agglomeration.

Region	Cities	2000 (RMB, Billion)	2018 (RMB, Billion)	2018–2000 (RMB, Billion)	Change Rate (%)
Central city	Zhengzhou	9.816	19.346	9.531	97.097
Metropolitan area	Kaifeng	7.626	12.206	4.580	60.058
	Jiaozuo	6.558	8.941	2.384	36.353
	Xuchang	5.666	10.260	4.593	81.062
	Xinxiang	10.947	17.655	6.708	61.277
Hinterland	Anyang	9.984	15.633	5.649	56.581
	Bengbu	7.586	11.969	4.382	57.764
	Bozhou	10.660	16.878	6.217	58.321
	Fuyang	13.179	20.493	7.315	55.505
	Handan	13.909	27.222	13.313	95.715
	Heze	14.437	25.341	10.904	75.528
	Hebi	2.783	4.627	1.845	66.295
	Huaibei	3.626	5.892	2.266	62.493
	Jiyuan	4.462	4.276	−0.186	−4.169
	Jincheng	25.239	19.377	−5.863	−23.230
	Liaocheng	9.675	18.378	8.703	89.953
	Luoyang	46.870	31.899	−14.971	−31.942
	Luohe	3.160	5.500	2.340	74.051
	Nanyang	64.349	52.009	−12.341	−19.178
	Pingdingshan	13.685	16.445	2.760	20.168
	Puyang	4.707	8.973	4.266	90.631
	Sanmenxia	29.941	21.160	−8.781	−29.328
	Shangqiu	12.729	21.940	9.211	72.362
	Xinyang	44.566	35.267	−9.299	−20.866
	Xingtai	16.427	26.594	10.167	61.892
	Suzhou	12.390	19.797	7.407	59.782
	Yuncheng	19.755	28.344	8.588	43.473
	Changzhi	26.581	30.164	3.584	13.483
	Zhoukou	14.816	24.220	9.404	63.472
	Zhumadian	24.252	29.287	5.035	20.761
Total		490.380	590.091	99.711	20.333

**Table 5:** Intensity and change of ESV spatially transferred from the cities to Zhengzhou.

Region	City	2000 (RMB, 10,000/km^2^)	2018 (RMB, 10,000/km^2^)	2018–2000 (RMB, 10,000/km^2^)
Central city	Zhengzhou	/	/	/
Metropolitan area	Kaifeng	82.98	132.82	49.84
	Jiaozuo	165.61	225.80	60.20
	Xuchang	129.89	235.18	105.30
	Xinxiang	132.50	213.70	81.20
Hinterland	Anyang	49.97	78.24	28.27
	Bengbu	4.99	7.87	2.88
	Bozhou	13.56	21.47	7.91
	Changzhi	57.69	65.47	7.78
	Fuyang	16.78	26.09	9.31
	Handan	24.61	48.17	23.56
	Hebi	14.41	23.96	9.55
	Heze	33.35	58.54	25.19
	Huaibei	3.8	6.17	2.37
	Jincheng	147.85	113.51	−34.34
	Jiyuan	36.39	34.87	−1.52
	Liaocheng	10.9	20.7	9.8
	Luohe	23.42	40.76	17.34
	Luoyang	254.85	173.45	−81.4
	Nanyang	147.85	119.49	−28.35
	Pingdingshan	130.02	156.24	26.22
	Puyang	11.43	21.79	10.36
	Sanmenxia	47.89	33.84	−14.05
	Shangqiu	30.36	52.33	21.97
	Suzhou	10.13	16.19	6.06
	Xingtai	16.48	26.69	10.2
	Xinyang	45	35.61	−9.39
	Yuncheng	38.86	55.75	16.89
	Zhoukou	55.6	90.89	35.29
	Zhumadian	59.55	71.92	12.37

**Table 6:** ESV spatial transfer amount and its changes in the Central Plains Urban Agglomeration from 2000 to 2018.

Region	City	2000 (RMB, Billion)	2018 (RMB, Billion)	2018–2000 (RMB, Billion)	Change Rate (%)
Central city	Zhengzhou	/	/	/	/
Metropolitan area	Kaifeng	3.153	4.507	1.354	42.943
	Xinxiang	5.441	7.94	2.499	45.929
	Jiaozuo	2.499	2.759	0.26	10.404
	Xuchang	1.99	3.432	1.442	72.462
Subtotal		13.083	18.638	5.555	42.460
Hinterland	Hebi	0.633	0.94	0.307	48.499
	Luohe	0.78	1.253	0.473	60.641
	Huaibei	0.976	1.404	0.428	43.852
	Jiyuan	1.363	0.824	−0.539	−39.545
	Puyang	1.485	2.775	1.29	86.869
	Bengbu	3.128	4.371	1.243	39.738
	Liaocheng	4.524	8.435	3.911	86.450
	Anyang	4.743	6.6	1.857	39.152
	Bozhou	5.23	7.416	2.186	41.797
	Suzhou	6.534	9.432	2.898	44.353
	Shangqiu	6.798	10.993	4.195	61.709
	Fuyang	7.153	9.932	2.779	38.851
	Pingdingshan	7.558	7.129	−0.429	−5.676
	Handan	7.74	15.098	7.358	95.065
	Heze	8.171	13.598	5.427	66.418
	Zhoukou	8.483	12.723	4.24	49.982
	Xingtai	9.845	14.593	4.748	48.228
	Yuncheng	12.805	16.012	3.207	25.045
	Zhumadian	17.067	16.791	−0.276	−1.617
	Jincheng	18.038	9.134	−8.904	−49.362
	Changzhi	19.375	17.523	−1.852	−9.559
	Sanmenxia	22.81	10.417	−12.393	−54.331
	Xinyang	38.892	21.93	−16.962	−43.613
	Luoyang	41.56	18.994	−22.566	−54.297
	Nanyang	62.697	37.806	−24.891	−39.700
Subtotal		318.388	276.123	−42.265	−13.275
Total		331.471	294.763	−36.71	−11.075

**Table 7:** The ESV spatial transfer amount into Zhengzhou and its change from 2000 to 2018.

Region	City	2000 (RMB, Billion)	2018 (RMB, Billion)	2018–2000 (RMB, Billion)	2000–2018 (%)
Metropolitan area	Kaifeng	0.140	0.120	−0.020	−14.286
	Xinxiang	0.194	0.206	0.012	6.186
	Jiaozuo	0.076	0.026	−0.050	−65.789
	Xuchang	0.032	0.042	0.010	31.250
Subtotal		0.442	0.394	−0.048	−10.860
Hinterland	Luoyang	1.264	0.140	−1.124	−88.924
	Pingdingshan	0.010	0.000	−0.010	−100.000
Subtotal		1.274	0.140	−1.134	−89.011
Total		1.716	0.534	−1.182	−68.881

**Table 8:** ESV spatial transfer radius of cities in the Central Plains Urban Agglomeration from 2000 to 2018.

Region	City	2000 (km)	2018 (km)	2018–2000 (km)	Change Rate (%)
Central city	Zhengzhou	/	/	/	/
Metropolitan area	Jiaozuo	28.30	25.47	−2.83	−10.02
	Xuchang	28.52	27.83	−0.69	−2.40
	Xinxiang	46.69	44.41	−2.28	−4.88
	Kaifeng	44.91	42.44	−2.47	−5.51
Hinterland	Luohe	42.05	40.40	−1.65	−3.93
	Jiyuan	44.59	35.41	−9.18	−20.59
	Hebi	48.29	45.65	−2.64	−5.47
	Pingdingshan	55.55	49.21	−6.33	−11.40
	Anyang	70.98	66.91	−4.06	−5.72
	Jincheng	80.47	65.35	−15.12	−18.79
	Puyang	83.03	82.21	−0.82	−0.99
	Zhoukou	89.99	86.20	−3.79	−4.21
	Luoyang	93.04	76.24	−16.80	−18.05
	Shangqiu	109.02	105.60	−3.42	−3.14
	Heze	114.03	111.04	−2.99	−2.63
	Huaibei	116.79	109.87	−6.93	−5.93
	Zhumadian	123.33	111.32	−12.01	−9.74
	Handan	129.19	128.98	−0.21	−0.16
	Yuncheng	132.25	123.47	−8.78	−6.64
	Changzhi	133.51	119.19	−14.32	−10.73
	Bozhou	143.09	135.41	−7.68	−5.36
	Liaocheng	148.45	147.07	−1.37	−0.93
	Nanyang	150.03	129.59	−20.44	−13.62
	Fuyang	150.43	142.15	−8.28	−5.51
	Sanmenxia	159.00	127.82	−31.18	−19.61
	Xingtai	178.05	170.37	−7.68	−4.31
	Bengbu	182.45	171.70	−10.76	−5.89
	Suzhou	184.99	175.83	−9.16	−4.95
	Xinyang	214.17	180.79	−33.39	−15.59

## Data Availability

Data is contained within the article. For detailed information of each part, please contact the corresponding author.
